# Post-Fracture Osteolysis of the Superior Pubic Ramus

**DOI:** 10.7759/cureus.23271

**Published:** 2022-03-17

**Authors:** Zhanfeng Mo, Qian Lu, Xuesheng Jiang

**Affiliations:** 1 Orthopaedics, Huzhou Central Hospital, Huzhou, CHN

**Keywords:** conservative treatment, radiotherapy, osteoporosis, post-fracture, pubic osteolysis

## Abstract

Post-fracture osteolysis of the pubic bone is a rare entity characterized by destructive changes in the pubic bone. We report a case of a 70-year-old woman who presented with a four-month history of left-sided groin pain radiating to the left hip. The radiographs showed osteolysis of the left superior pubic ramus, mimicking a malignant lesion. Histological examination showed no evidence of malignancy. After eight weeks of conservative treatment, the pain was significantly relieved. Post-fracture osteolysis may simulate malignancy; physicians should be aware of that to avoid unnecessary invasive procedures.

## Introduction

Post-fracture osteolysis of the pubic bone is rare, which was first described in 1978 [1]. To date, a few cases have been reported around the world. It is characterized by destructive changes in the pubic bone [1-3]. The underlying mechanisms of this condition remain unclear. It is a benign lesion, and it mostly occurs in patients with osteoporosis or patients after pelvic radiotherapy [4-6]. We herein report a case of an old woman with post-fracture osteolysis of the pubic bone mimicking a malignancy.

## Case presentation

A 70-year-old woman presented with a four-month history of left-sided groin pain after a fall; the pain was progressive, radiating to the left hip, causing a moderate limp. She had no fever, cough, night sweats, or weight loss. She had not received any treatment before her first clinic visit. She reported natural menopause at 52 years of age and had never used any hormone replacement therapy. She had no history of tumors or tuberculosis. Physical examination showed obvious tenderness on the left side of the pubic symphysis and limited walking distance due to left hip pain.

**Figure 1:**
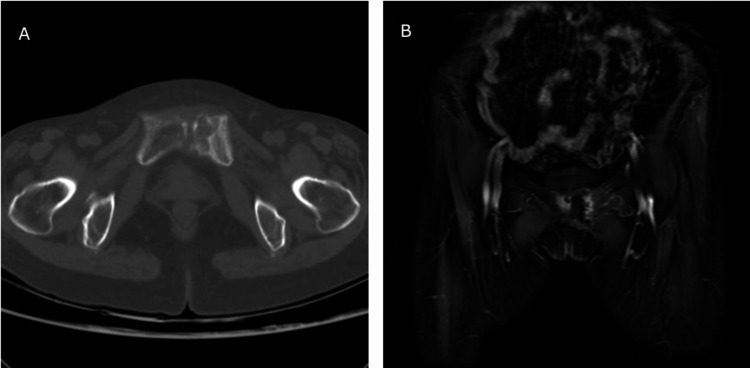
Imaging examination of the pubic bone (A) CT scan showing destruction and fracture of the left superior pubic ramus. (B) MRI showing hypointense on T1-weighted images, isointense, and hyperintense on T2-weighted images.

**Figure 2:**
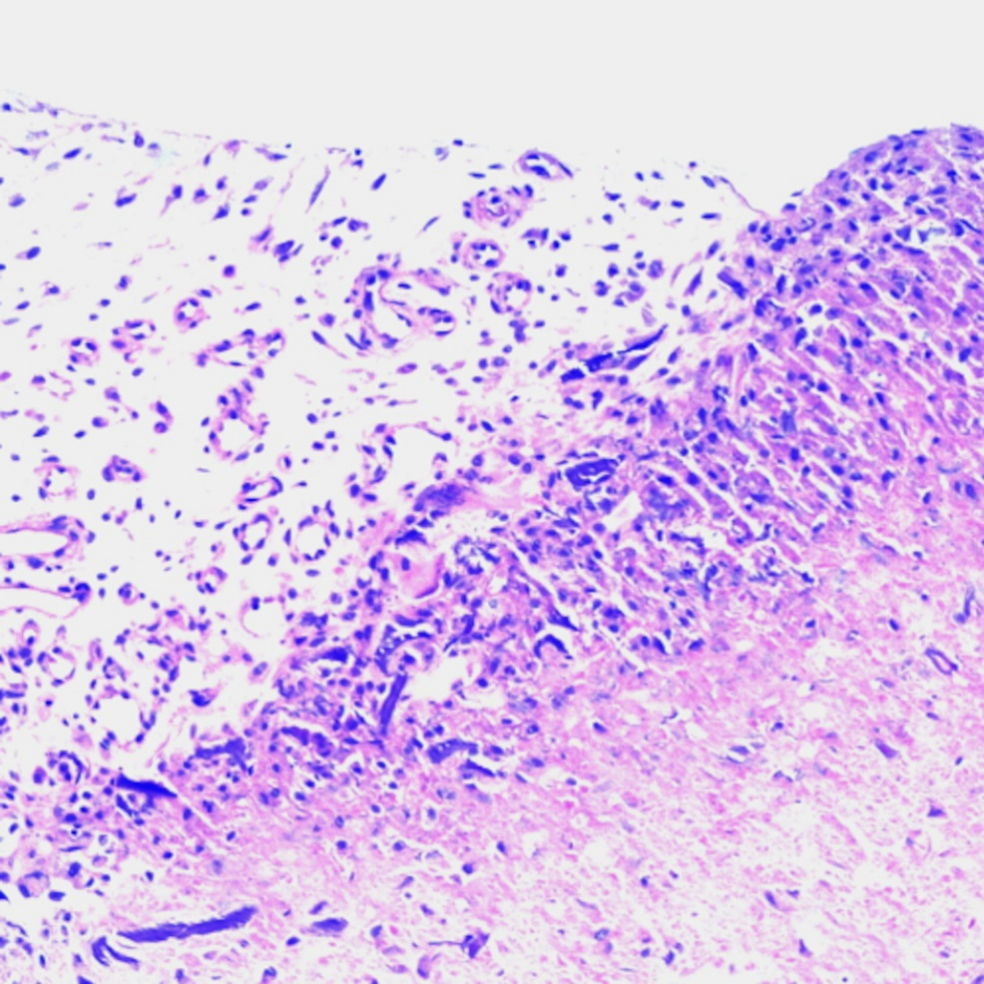
Histologic examination showing no evidence of malignancy. A biopsy demonstrating benign tissue composed of necrotic tissue, fibrous tissue, and blood vessels.

Laboratory examinations showed leukocyte 4.1×10^L (normal range 3.5-9.5×10^L), erythrocyte 3.62×10^12/L (normal range 3.8-5.1×10^12/L), platelets 83×10^L (normal range 120-350×10^9/L), hemoglobin 112g/L (normal range 115-150g/L), erythrocyte sedimentation rate (ESR) 15mm/h (normal range 0-20mm/h), serum calcium 2.16mmol/L (normal range 2.17-2.75mmol/L), serum alkaline phosphatase 78.5U/L (normal range 50-135U/L), aspartate aminotransferase 17.3U/L (normal range 13-35U/L), alanine transaminase 13.2U/L (normal 7-40U/L), serum creatinine 79.5μmol/L (normal range 44-132μmol/L), urea nitrogen 4.79mmol/L (normal range 3.2-7.1mmol/L). Computed tomography (CT) scan revealed obvious destruction and fracture of the left superior pubic ramus, and no abnormality was found on the bilateral hip joint (Figure 1A). Magnetic resonance imaging (MRI) showed bone destruction of the left superior pubic ramus, muscle, and subcutaneous soft tissue were edematous, the lesion was mixed isointense and hyperintense on T2-weighted images, and was hypointense on T1-weighted images (Figure 1B). Dual-energy X-ray absorptiometry (DXA) was used to evaluate the patient’s bone mineral density (BMD), and the T-score was -2.6 SD, which confirmed the diagnosis of osteoporosis. Abdominal ultrasound showed splenomegaly and gallbladder polyp. A biopsy specimen was taken from the lesion, and histologic examination showed necrosis, fibrous tissue proliferation, and angiogenesis; there was no evidence of malignancy (Figure 2).

The patient received conservative treatment. Since there was no specific medicine for osteolysis, she received medications with oral bisphosphonates, vitamin D, calcium for osteoporosis, and non-steroidal anti-inflammatory drugs (NSAIDs) for pain relief. She was given a pelvic binder and was advised to reduce activity and walk with a stick for 4 to 6 weeks. Eight weeks after the treatment, the patient’s pain was significantly relieved, the visual analog scale (VAS) was decreased from 7 to 2, and then the patient was lost to follow-up.

## Discussion

Post-fracture osteolysis is a rare benign lesion. There are only a few reports about it. The typical radiologic characteristics are similar to malignant bone tumors, which may lead to misdiagnosis and unnecessary invasive operations [4,6]. In addition to cases of pubic osteolysis, there are also a few cases of distal clavicle osteolysis, even post-traumatic spine or femoral neck osteolysis [7-10]. The pathogenesis of the osteolytic process remains unclear; the current hypothesis suggests that it may be related to the inefficient immobilization of the bone fractures, which lead to bone non-union and resorption, or radiation-induced osteonecrosis [1, 2,11]. Pubic osteolysis often occurs in postmenopausal women with osteoporosis after trauma or in patients with a history of radiotherapy [1,2]. Clavicular osteolysis mostly happens after trauma and has nothing to do with age or gender [8,12]. Some patients with osteolysis of the lateral clavicle have ipsilateral pupil enlargement, which suggests that the autonomic nervous dysfunction may lead to local ischemia and osteolysis [12,13]. In addition, osteoclasts’ overactivity, vascular compromise, and hyperplastic tissue compression may be related to osteolysis [12-14].

Patients mainly complain of groin pain and limited flexion and extension of the left hip, which causes a limp a severe fracture can cause pelvic deformity [2,6]. Imaging examination is helpful for the diagnosis of osteolysis. Osteoporosis, fracture, and separation of pubic symphysis can be seen on X-ray, but in the early stage, the fracture may not be found [4,6]. CT and MRI are both the keys to the diagnosis of post-fracture pubic osteolysis. CT scanning can show fracture, destruction, and necrosis of the pubic bone. It may also show a separation of the pubic symphysis in some patients. MRI can show soft tissue swelling, bone marrow edema, and bone fracture. Sometimes MRI will reveal a linear hyperintense line, which is helpful to distinguish between a benign pubic fracture and a malignant mass [4]. The lesion is mixed isointense and hyperintense on T2-weighted images and is hypointense or isointense on T1-weighted images [2]. Laboratory tests for inflammation, serum calcium, phosphate, and serum alkaline phosphatase are usually in the normal range, showing no evidence of inflammation or bone metabolic diseases. Histologic examination showed fibrocartilage and connective tissue hyperplasia, and there is no evidence of malignancy [2,6]. Post-fracture pubic osteolysis is a self-healing disease; it can be treated conservatively with bed rest, analgesic, calcium tablet, and vitamin D. The bone healing time is reported to be 4-5 months [5].

## Conclusions

Post-fracture pubic osteolysis can mimic a malignancy; it is little-known to physicians. MRI and CT scans, as well as histologic examination, are important for the diagnosis of the disease; conservative treatment can achieve good results. Physicians should be aware of that to avoid unnecessary surgical treatment.
